# Physician Orders for Life Sustaining Treatment in US Nursing Homes: A Case Study of CRNP Engagement in the Care Planning Process

**DOI:** 10.1155/2014/761784

**Published:** 2014-04-29

**Authors:** Gerald A. Hartle, David G. Thimons, Joseph Angelelli

**Affiliations:** ^1^Heritage Valley Family Practice, 2620 Constitution Boulevard, Beaver Falls, PA 15010, USA; ^2^School of Nursing and Health Sciences, Robert Morris University, 6001 University Boulevard, Moon Township, PA 15108, USA

## Abstract

This case study describes changes in Physician Orders for Life Saving Treatment (POLST) status among long-stay residents of a US nursing home who had a certified registered nurse practitioner (CRNP) adopt the practice of participating in nursing home staff care plan meetings. The CRNP attended a nonrandomized sample of 60 care plan meetings, each featuring a review of POLST preferences with residents and/or family members. Days since original POLST completion, Charlson Comorbidity Index score, number of hospitalizations since index admission, and other sociodemographic characteristics including religion and payer source were among the data elements extracted via chart review for the sample as well as for a nonequivalent control group of 115 residents also under the care of the medical provider group practice at the nursing home. Twenty-three percent (*n* = 14) of the 60 care conferences attended by the CRNP resulted in a change in POLST status after consultations with the resident and/or family. In all cases, POLST changes involved restated preferences from a higher level of intervention to a lower level of intervention. Fifty-nine percent of the CRNP-attended conferences resulted in the issuance of new medical provider orders. CRNP participation in care conferences may represent a best practice opportunity to revisit goals of care with individuals and their family members in the context of broader interprofessional treatment planning.

## 1. Introduction

Over two decades ago, research using prospective study methods examined end-of-life planning practices and found 25 percent of nursing home residents received care that was inconsistent with previously expressed wishes [1]. Geriatric treatment protocols now suggest that advance care planning should be thoroughly discussed upon admission to nursing homes and revisited routinely [2], as one-fourth of all deaths within the United States occur in a nursing home setting and 70 percent occur in hospitals [3]. Best practices for establishing and honoring current treatment preferences among long-stay nursing home residents remains an area in need of attention, as failure to update care plans and respond to changing goals of care can result in unwanted treatment and unnecessary transfers to acute care facilities [4]. This paper documents a case study of CRNP participation in nursing home resident care plan meetings as an important step in delivering person-centered end-of-life care.

For most institutionalized persons living with dementia and/or other multiple chronic conditions, the decision to forego hospitalization is not made until death is imminent and frequent hospitalizations are common near the end-of-life [5]. The small number of nursing home residents with “do not hospitalize” orders is an indication of the limited use of end-of-life preference documentation for the US nursing home population [6]. However, studies across multiple settings have shown that clinicians are often not educated or equipped to have open discussions about the value of medical interventions [7]. In nursing homes, medical directors perceive the lack of information and support to residents and families around end-of-life care and the lack of familiarity with residents by covering doctors as the most important causes of unwanted and preventable hospitalizations [8].

The Physician Orders for Life Sustaining Treatment (POLST) program was launched in Oregon in 1991. Today, 41 states have developed or are developing POLST programs [9]. The POLST is designed to follow patients through all care settings including hospitals, nursing homes, home care, and hospice. The POLST is a one-page current treatment document to be completed by the health care professional and the patient or patient surrogate. In the US, the document may be completed by other qualified and trained health care professionals in addition to physicians (i.e., nurse practitioners, physician's assistants, or social workers) [10]. The intent of the POLST is to provide a mechanism for health care professionals to turn the patient's or family's goals for care into action by establishing standing orders for* current* medical treatment (as compared to an advanced directive, which is intended to provide instructions for* future* treatment). The POLST clearly and specifically identifies various modalities of care, including the decision to resuscitate or not to resuscitate (DNR), comfort measures only/limited additional treatment/full treatment, use of antibiotics, and the desire for medically administered nutrition.

The use of patient-centered care planning at the end-of-life has been identified by the Institute for Healthcare Improvement (IHI) as a key component in decreasing hospitalization, though decreased hospitalizations should not be considered a goal in and of itself in advance care planning [11]. In 2011, the National Institute of Nursing Research Summit recommended:

**…further study of patients' and family members' change in perceptions of advance care plans over time, diagnosis, and illness-related changes. Further, although the role of the surrogate has received some attention in research, more research is needed in order to fully understand the influence of surrogates in end-of-life decision making. Continued focus on developing educational interventions aimed at patients, families, and providers (cross-disciplinary training) is needed as well as development of a standardized definition of advance care planning in different settings. [12]**

Although the recommendation is for end-of-life goals of care to be discussed early and routinely updated, there are no criteria specific to how often or when updates to the POLST should be done. Some practitioners suggest reviews should be done with change in condition, upon family or resident request, during regularly scheduled care conferences, and/or at readmission from acute care [13]. In US nursing homes where the POLST is used, it is typically completed at the time of admission by a social worker (if no POLST form accompanies the resident at admission). A recent survey of 115 nursing homes in Pennsylvania (where the case study nursing home is located) found only 40% of homes using POLST, and among those, the social worker was identified as the prime initiator of POLST conversations 85.7% of the time [14]. The physician or CRNP later signs and confirms the order. However, research has documented the extent to which nursing home residents and their surrogates are often overwhelmed at the time of admission and later acknowledge never recalling filling out a POLST [2].

Care conferences are a valuable time for health care professionals to listen to what the individual considers worthwhile living and respect their wishes for end-of-life treatment. The conferences are viewed as an opportunity to bring all involved to the “same page” regarding the situation and care [15], an important goal as the general public has been shown to have an inflated perception of CPR effectiveness [16]. Fewer than 5 percent of frail nursing home residents survive an attempted CPR long enough to return to the nursing home [17]. Among long-stay residents, a regular review of a nursing home resident's condition and her or his POLST status should reflect potentially changing goals of care. CRNPs attending care plan meetings (not currently standard practice in most US nursing homes) may positively influence the complete and timely attention to POLST goals of care.

## 2. Materials and Methods

The POLST Conversation Documentation Tool was used to guide the intervention as it provides an effective framework for patients and their families to make informed decisions [18]. In nursing homes, standard practice is for social service professionals, nurses, therapists, administrative staff, and families to meet quarterly with each resident to review medical condition(s) and treatment plans. Physician and/or CRNP participation in quarterly care plan meeting is rare in US nursing homes. In the nursing home in which this case study was conducted it was not standard practice for the POLST to be reviewed at care plan meetings. This study was an attempt to document a structured approach to reviewing the POLST with CRNP input at care plan meetings.

The medical group practice (averaging 175 nursing home residents at the time of the case study) established a nonrandomized sample of 60 residents to expand the care plan meetings to include CRNP participation and a review of the POLST. Attendance in the care conference was initiated as care plan meeting schedules were aligned with the CRNP's availability (beginning November 2012 and continuing through February 2013). The intervention involved a more extensive chart review, a discussion of each resident's condition with various professional representatives, and a proactive interaction with residents and their families regarding POLST as outlined by the POLST Conversation Documentation Tool. POLST conversations were initiated by the CRNP during quarterly care plan meetings as well as ad hoc care plan meetings organized following ER visits or hospitalizations. Thus, it was more likely for nursing home residents to be included in the case study group simply by virtue of returning to the nursing home from the hospital.

The POLST Documentation Tool calls for provider meetings at the bedside with nursing home residents unable to attend a care plan meeting and a review of the POLST in person or by phone with family members unable to participate in the on-site care plan conversation. If the care plan meeting and subsequent resident or family discussion results in a POLST status change, a new POLST is prepared and placed in the chart. Also, the medical chart is updated with a progress note to reflect results of a physical assessment, attendees of the conference, the POLST status pre/postcare conference, any new or changed medical orders, and the length of time taken for the process.

Sociodemographic data including age, religion, gender, and payer source were extracted via chart review, also extracted were the number of comorbidities, number of hospitalizations within the last year, number of emergency department (ED) visits not resulting in a hospital visit, original and most recent dates of admission to the nursing home, date of original POLST completion and whether the original POLST was completed by a social worker versus a physician or CRNP.

Comorbidities were measured using the Charlson Comorbidity Index (CCI) [19]. The CCI is a valid and reliable method for assessing comorbidities in clinical research [20]. The use of data obtained from medical records is a standard approach, as manual abstraction of data from medical records is a routine and efficient method of data collection for clinical databases, audits, and clinical research. Its advantages are accessibility, depth of information, cost, and flexibility in the time the study is conducted [18, 21]. Chart extraction for all 175 nursing home residents was conducted post hoc by a trained assistant blinded to the purpose and scope of the study. Institutional review board (IRB) approval was obtained through the appropriate organizational process.

Data elements were analyzed using SPSS 20 to determine to what extent the 60 residents who had the CRNP participate in a care plan meeting were dissimilar to the other 115 nursing home residents in the group practice [22]. Among the 60 residents receiving the nonrandomized intervention, *t*-test and chi-square tests were used post hoc to explore potentially significant differences in means and expected distributions between those whose care conference resulted in a change of POLST status versus those who did not elect a change in POLST. Differences on continuous variables were analyzed using *t*-tests (Mann-Whitney *U* for skewed distributions).

## 3. Results

Among all 175 nursing homes residents under the care of the group practice at the time of the intervention, mean time since original admission to the nursing home was 886 days and mean time since POLST completion was 755 days. The mean number of hospitalizations since original nursing home admission was 0.88 (standard deviation = 1.22) and the mean number of ED visits not resulting in hospital admission was 0.72 (standard deviation = 1.41).

In comparing the 60 nursing home residents in the case study to the 115 nursing home residents receiving usual care (Table 1), the case study nursing home residents were significantly more likely to be female (83% versus 67%) and had significantly higher mean comorbidity scores when compared to the other 115 residents (4.95 versus 4.34). The 60 case study residents were also different in religious composition, with a significantly higher proportion characterized as “no religion” (19%) versus the usual treatment group (10%). Also, the 60 case study nursing home residents had significantly more mean ED visits not resulting in hospital admission prior to the care conference when compared to the 115 in the usual care group (1.1 versus 0.5). There were no statistically significant differences in age, days since original POLST completion, or days since original/most recent nursing home admission between the 60 cases and 115 nursing home residents in the usual care group, nor any difference in the payer source distribution of each group. The percentage in each group who had a social worker complete the original POLST was similar (73% versus 78%). 

Twenty-three percent (*n* = 14) of the 60 care conferences attended by the CRNP resulted in a change to the POLST goals of care (Table 2). Among the 14 who elected to change their POLST status, a significantly higher proportion had self-reported as “no religion” when compared to the 46 with no change in POLST status. There were no significant differences in our measures of other sociodemographic characteristics or other previous health care utilization among those whose POLST status changed compared to others with no change in POLST status. More than half (59%) of all care conferences attended by the CRNP resulted in new orders such as lab work, dietary changes, and specialty consults.

Table 3 shows the POLST status of nursing home residents prior to and following CRNP-attended care conference, along with the distribution of the standing POLST status among the 115 other nursing home residents in the group practice. The distribution of POLST order categories at baseline is reported for each group, with 8 percent of the 115 comparison group having no initial POLST on record. Of the 60 residents who had the CRNP attend their care conference, all changes in POLST goals of care were to a lesser level of intervention. The total number of residents or their surrogates electing* comfort care status with DNR orders *increased from 21 to 30.

## 4. Discussion

A review of our care conference case study illustrates the possible importance of direct CRNP participation in regular care planning meetings as a method of revisiting POLST goals of care with long-stay nursing home residents and their families. The structured involvement of a CRNP in care plan meetings seemed to promote an individualized approach to POLST goals of care that had not occurred in previous interprofessional meetings led by nursing and social work staff. Decisions by the 23 percent of residents and/or family members to elect less aggressive treatment options on the POLST (from “full treatment” to “limited treatment”) were made after personal consultations with the CRNP. Worth noting is the finding that for 77 percent of the cases the POLST options for current treatment remained unchanged following the care conference and family consultations, suggesting an important degree of stability in POLST preferences over time for this sample of long-stay nursing home residents.

Having a CRNP play a more active role in the care planning process may have created an opportunity to be more responsive to the broader care needs of residents, as evidenced by the majority of residents who received new or adjusted medical orders (treatments that might not otherwise have been ordered in the absence of active involvement by the CRNP). In addition to the regularly scheduled quarterly care plan meetings, care conferences were conducted after each hospital admission and/or any change in condition, a selection artifact that may explain the higher comorbidity scores and higher historical rates of ED visits pre-care conference in the 60 residents who had the CRNP attend a care plan meeting during the study timeframe.

While the goal of POLST use should not be to prevent hospitalizations per se, it worth noting that research has demonstrated how nursing homes that have higher facility-level rates of DNR orders have lower rates of hospitalization [23]. At present, CRNP participation in the care planning process is rare in U.S. nursing homes, yet it represents an important marker for establishing DNR preferences, particularly now as both hospitals and nursing homes are focused on lowering rates of preventable acute care transfers. Indeed, many of the various efforts underway to reduce rehospitalizations from nursing homes involve greater attention to advance care planning, including having the CRNP and/or physicians initiate and sustain goals of care conversations using tools featured in the INTERACT 3.0 quality improvement program [24]. Evidence of an individualized and timely CRNP review of POLST status is important also for nursing homes in terms of meeting regulatory requirements clarified in an October 18, 2013 memorandum from the Centers for Medicare and Medicaid Services (CMS), which stipulated that nursing homes must not establish nor implement facility-wide “no CPR” policies for their residents, as this does not comply with each resident's right to establish individualized goals of care [25].

Research on the value of POLST has shown nursing home residents with POLST forms were more likely to have treatment preferences documented as medical orders than those who did not, but there were no differences in symptom management [26]. POLST orders restricting medical interventions were associated with less use of life-sustaining treatment, a finding that suggests that the POLST offers significant advantages over traditional methods to communicate preferences about life-sustaining treatments for individuals with serious illness. Another research work has confirmed that the POLST program facilitates documentation of a range of current treatment preferences and is associated with a decrease in rates of unwarranted hospitalizations consistent with the person's individualized goals of care [27–29]. Ninety-seven percent of POLST users reflected a preference to restrict hospitalization or ICU care, whereas only 14% of non-POLST users had reflected hospitalization preferences [26]. However, using POLST preference to restrict hospitalization or ICU care should never be the motivating factor for its use. Great care must be taken by CRNPs and medical providers to avoid introducing their own biases that may unwittingly guide patients or proxies toward less intensive treatment.

This case study had several limitations, including using a nonequivalent design and being conducted within a single group practice in one nursing home with a modest panel of 60 nonrandomized nursing home residents. CRNP participation in care plan meetings has since been routinized as a standard practice for all 175 nursing home residents receiving care via the medical group. Future research will explore the possible effects of POLST status changes on rates of hospitalization from the nursing home in the context of broader efforts to reduce avoidable hospitalizations.

## 5. Conclusion

Care plan meetings attended by CRNPs to review POLST goals of care in nursing home settings can be a key aspect of person-centered geriatric treatment near the end-of-life. As efforts spread to individualize care preferences, improve care transitions, and lower overall health care costs for elders in nursing homes, payers and policymakers should recognize the value of consistent advance care planning and establish adequate reimbursement mechanisms for these critical but time-consuming resident and family conversations. Clinician researchers should continue to explore the role of timing as a factor in POLST decisions. Should CRNPs attend care plan meetings quarterly or semiannually? How do other disciplines value CRNP participation in the care plan meetings? How can social workers play a more active role in collaboration with residents, families, physicians, and CRNPs in revisiting POLST treatment plans? Is there improved resident and family satisfaction with enhanced CRNP participation? As the system evolves toward more value-driven methods of coordinated care, the importance of timely and consistent review of goals of care should emerge as a high priority for all those with a stake in fostering person-centered care.

## Figures and Tables

**Table 1:** Demographic and health care utilization characteristics of nursing home residents in provider group practice, by CRNP participation in care conference.

	CRNP attended care conference *n* = 60 Mean (standard deviation)	Other nursing home residents under care of provider group practice *n* = 115 Mean (standard deviation)
Age in years	74.6 (14.6)	72.0 (14.3)
% Female	83%*	67%
Religion		
% Catholic	25%	33%
% Protestant	44%	53%
% Another religion	7%	3%
% No religion	19%*	10%
% Unknown religion	5%	1%
Payer source		
Medicaid FFS	63%	58%
Medicare FFS	7%	17%
Medicaid managed care	18%	18%
Medicare managed care	12%	8%
Charlson comorbidity index	4.95 (1.92)*	4.34 (1.72)
% Social worker completing original POLST	73%	78%
Days since original POLST completion	720 (700)	772 (851)
Days since original nursing home admission	863 (806)	899 (834)
Days since last nursing home admission	610 (592)	590 (658)
Number of hospital admissions	1.02 (1.40)	0.80 (1.11)
Number of emergency department visits not resulting in hospital admission	1.10 (1.91)*	0.52 (1.01)

*P < 0.05.

**Table 2:** Demographic and health care utilization characteristics among nursing home residents who had CRNP participate in care conference, by POLST change in status versus no POLST change in status.

	POLST change in status *n* = 14 Mean (standard deviation)	No POLST change in status *n* = 46 Mean (standard deviation)
Age in years	75.7 (13.3)	74.3 (15.2)
% Female	71	87%
Religion		
Catholic	14%	29%
Protestant	21%	51%
Another religion	7%	7%
No religion	43%*	11%
Unknown	15%	2%
Payer source		
Medicaid FFS	86%	57%
Medicare FFS	7%	7%
Medicaid managed care	7%	24%
Medicare managed care	0%	13%
Charlson comorbidity index	4.50 (1.93)	5.11 (1.95)
% Social worker completing original POLST	79%	72%
Days since original POLST completion	707 (542)	724 (746)
Days since original nursing home admission	839 (587)	871 (878)
Days since last nursing home admission	712 (572)	572 (616)
Number of hospital admissions (precare conference)	0.56 (0.96)	1.18 (1.51)
Number of emergency department visits not resulting in hospital admission (precare conference)	1.18 (1.72)	1.06 (2.00)

*P < 0.05.

**Table 3:** POLST status of nursing home residents prior to and following CRNP-attended care conference versus other nursing home residents in the provider group practice.

POLST status	Number of residents prior to CRNP-attended care conference *n* = 60 Number of cases (% to total)	Number of residents following CRNP-attended care conference *n* = 60 Number of cases (% of total)	Number of other nursing home residents in CRNP group practice *n* = 115 Number of cases (% of total)
Full treatment/CPR	16 (27%)	13 (22%)	36 (31%)
Limited treatment/CPR	1 (1%)	0	0
Limited treatment/DNR	22 (37%)	17 (28%)	42 (37%)
Comfort care/DNR	21 (35%)	30 (50%)	29 (25%)
No POLST on record	0	0	8 (7%)

A chi-square test was performed to examine whether the distributional properties of the POLST status differed significantly pre- and post-care plan meeting. There were no significant differences.
